# An Organoselenium Compound Induces Death in *Cryptococcus gattii* Yeast by Apoptosis and Necrosis and Shows Antifungal Efficacy in *Galleria mellonella*

**DOI:** 10.1021/acsomega.6c02274

**Published:** 2026-07-14

**Authors:** Letícia Serafim da Costa, Daniel Felipe Freitas de Jesus, Larissa Dunkl Brujas, Isadora Maria de Oliveira, Rafael Wesley Bastos, Analy Salles de Azevedo Melo, Daniel Assis Santos, Hélio A. Stefani, Kelly Ishida

**Affiliations:** † Department of Microbiology, Institute of Biomedical Sciences, University of São Paulo (USP), 1374 Prof. Lineu Prestes Avenue, São Paulo, SP 05508-000, Brazil; ‡ Department of Pharmacy, School of Pharmaceutical Sciences, University of São Paulo (USP), 580 Prof. Lineu Prestes Avenue, São Paulo, SP 05508-000, Brazil; § Center of Biosciences, 28123 Federal University of Rio Grande do Norte (UFRN), 3000 Sen Salgado Filho Avenue, Natal, RN 59064-741, Brazil; ∥ Department of Medicine, Federal University of São Paulo (UNIFESP), 740 Botucatu Street, São Paulo, SP 04023-062, Brazil; ⊥ Institute of Biomedical Sciences, 28114 Federal University of Minas Gerais (UFMG), 6627 Pres. Antônio Carlos Avenue, Belo Horizonte, MG 31270-901, Brazil; # Brazilian National Institute of Science and Technology in Human Pathogenic Fungi (INCT-FUNVIR, Ribeirão Preto, SP 14049-900, Brazil

## Abstract

*Cryptococcus gattii* is an etiological agent of invasive pulmonary cryptococcosis in both immunocompetent and immunocompromised individuals. Current antifungal therapy is limited by toxicity, high cost, and emerging tolerance, supporting the need for new therapeutic alternatives. Although the organoselenium compound LQA_78 previously demonstrated antifungal activity against *Cryptococcus neoformans*, its effect on *C. gattii* remains unclear. Then, the aim of this work is to evaluate the antifungal activity of LQA_78 against *C. gattii* strains, its effects on virulence factors, and the underlying mechanisms of fungal cell death. Clinical isolates and morphotypes of *C. gattii* were subjected to susceptibility testing to determine inhibitory and fungicidal concentrations. Capsule thickness and permeability, melanin production, and laccase activity were assessed. Oxidative stress parameters, including reactive oxygen species (ROS) production, lipid peroxidation, glutathione (GSH) levels, and superoxide dismutase activity, were evaluated. Mitochondrial membrane potential, DNA fragmentation, and chromatin condensation were analyzed to investigate cell death pathways. *In vivo* efficacy was assessed using the *Galleria mellonella* infection model. LQA_78 inhibited fungal growth at concentrations comparable to fluconazole (2–32 μg/mL), increased capsule permeability without reduction in capsule thickness, and melanin production and laccase activity were significantly reduced. LQA_78, at higher concentrations (16–32 μg/mL), induced and increased plasma membrane permeability, lipid peroxidation, ROS accumulation, GSH depletion, reduced superoxide dismutase activity, mitochondrial dysfunction, DNA fragmentation, chromatin condensation, and phosphatidylserine externalization, indicating apoptosis- and necrosis-like cell death. In *G. mellonella*, nontoxic doses reduced the larvae mortality and fungal burden and increased hemocyte density. Therefore, the organoselenium LQA_78 demonstrates significant *in vitro* and *in vivo* antifungal activity against *C. gattii*, supporting its potential as a lead compound for cryptococcosis therapy.

## Graphical abstract

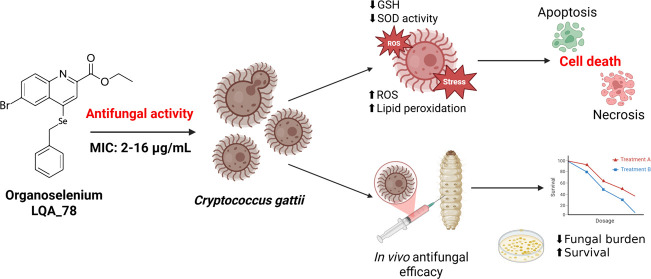

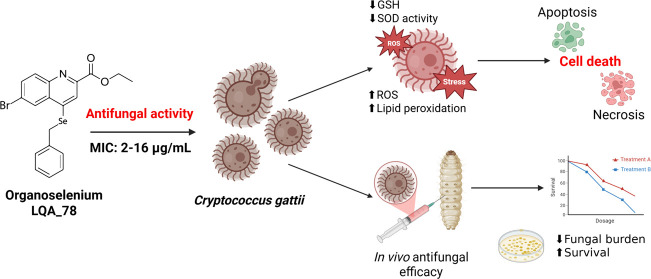

## Introduction

*Cryptococcus gattii* is a pathogenic fungus responsible for causing cryptococcosis, especially in immunocompetent patients, in contrast to *Cryptococcus neoformans* that usually affects immunocompromised hosts.^1^ Although *C. gattii* accounts for a smaller proportion of global cryptococcosis cases compared to *C. neoformans*, it is responsible for a significant number of infections in specific geographic regions, particularly in tropical and subtropical areas, representing approximately 11–33% of cryptococcosis.^2^ Globally, cryptococcosis is associated with an estimate of ∼180,000 deaths annually,^3^ primarily due to cryptococcal meningitis, and *C. gattii* contributes to this burden, often causing severe disease even in healthy individuals. Recently, the World Health Organization has published the Fungal Priority Pathogens List to Guide Research, Development and Public Health Action,^4^ and among other important fungal pathogens, *C. gattii* was cited due to its high mortality and lack of treatment options.

Cryptococcosis caused by *C. gattii* presents mostly in the host’s lungs, but it can also spread to the central nervous system, causing cryptococcal meningitis.^5^ These clinical manifestations are driven by *C. gattii* virulence factors, which play a critical role in the pathogenesis of cryptococcal infection. Key determinants of infection establishment include the polysaccharide capsule, cell wall components, melanin production, extracellular enzymes, and the ability to transition from typical yeast cells to titan cells.^6–10^ Furthermore, *C. gattii* is able to infect immunocompetent individuals due to its high ability to evade a host’s immune response.^11^

Treatment for cryptococcosis is based in drug therapy using a combination of three antifungals when available, such as amphotericin B (AMB), fluconazole (FLC), and 5-flucytosine (5-FC).^4,12^ In addition to therapeutic approaches, antifungal prophylaxis strategies are currently employed in specific high-risk populations, particularly among immunocompromised patients.^13^ However, despite these advances, significant limitations persist. The limited availability of treatment options, the high toxicity of AMB, and emerging of resistance and heteroresistance to FLC remain major concerns.^14–17^ These limitations continue to be highlighted in recent literature and were recognized by the World Health Organization, reinforcing the need for the development of new antifungal strategies.^4^

Due to this urgent need, one of the research pathways usually explored is the prospecting of new molecules. Organoselenium compounds have emerged as promising pharmaceutical options for different applications, including against infection diseases.^18^ Selenium-containing compounds such as ebselen and diphenyl diselenide have been shown to have antifungal activity against several fungal species such as *Candida albicans*, *C. neoformans*, *Aspergillus fumigatus*, and *Sporothrix brasiliensis*, highlighting the need for further studies and for taking organoselenium into consideration as a potential antifungal.^19,20^ In addition, selenium compounds have been described as nontoxic and safe in murine models and clinical trials,^21^ which comes in contrast with the high toxicity that standard antifungals show. Recently, our research group has demonstrated the antifungal activity and inhibition of virulence factors of a synthetic selenium compound (LQA_78, Figure 1) against *C. neoformans*.^22^ Thus, this present study aims to demonstrate the antifungal action of the LQA_78 compound against *C. gattii* isolates and its mechanisms to induce growth inhibition and cell death, using *in vitro* and *in vivo* experimental models, in order to add data and to enhance knowledge about organoselenium compounds as potential antifungal agents.

**Figure 1:**
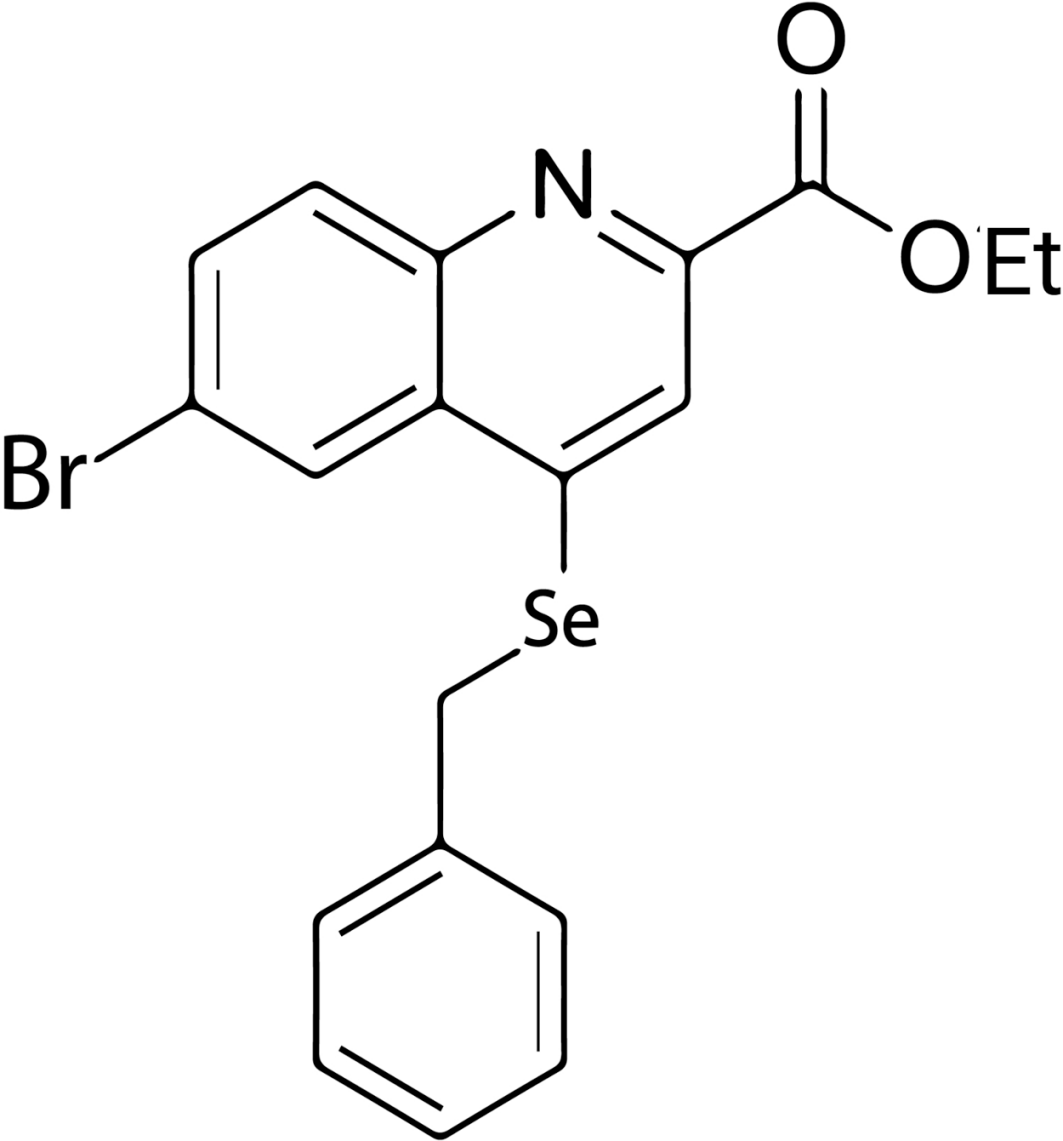
Chemical structure of LQA_78 (ethyl 4-(benzylselanyl)-6-bromoquinoline-2-carboxylate).

## Methods

### Microorganisms

*C. gattii* R265 (nonadapted, NA) was used in all experiments, along with a tebuconazole (TBZ)-adapted variant of the same strain (R265 A), which exhibits cross-resistance to clinical azoles due to overexpression of efflux pump genes and an enhanced capacity to acetylate fluconazole.^23,24^ In addition, five human clinical isolates previously identified as *C. gattii* ^25^ were included. Yeasts were maintained in brain heart infusion broth containing 20% glycerol at −80 °C and recovered on Sabouraud dextrose agar (SDA; Becton, Dickinson, Sparks, MD, USA) at 35 °C. Prior to each experiment, cultures were subcultured twice on the same medium for 48–72 h at 35 °C.

### Antifungal Drugs

Fluconazole (FLC) and amphotericin B (AMB) (Sigma-Aldrich, St. Louis, MO, USA) were dissolved in dimethyl sulfoxide (DMSO; Vetec, St. Louis, MO, USA) while 5-flucytosine (5-FC) was dissolved in sterile water. The stock solutions were prepared at least 100-fold higher than the maximum test concentration (e.g., 2560 μg/mL for FLC/5-FC and 1600 μg/mL for AMB) and stored at −20 °C.

### Organoselenium LQA_78

The compound LQA_78 was synthesized as previously described^26^ (Figure 1). The powders and stock solutions in DMSO were aliquoted and stored at −80 °C. The final concentration of DMSO in the work solution was ≤5%.

### Antifungal Activity of Organoselenium LQA_78

#### Planktonic Cells

The inhibitory effect of LQA_78 and standard antifungals was assessed using the broth microdilution technique.^27^ Compounds were tested against R265 (NA and A) and 5 clinical isolates of *C. gattii*. The lowest concentrations that inhibit fungal growth by 50% and 90% (IC_50_ and IC_90_, respectively) were visually determined after incubation for 48 h at 35 °C. *Candida parapsilosis* ATCC 22019 was used as a quality control strain in the assay. The criteria of interpretation for standard antifungals were based on^27,28^ considering the IC_50_ values for FLC and 5-FC while IC_90_ values for AMB. The minimum fungicidal concentration (MFC) was determined after subculture of yeasts treated with inhibitory concentrations and it was defined as the lowest concentration of the compound able to reduce the cell viability by >99.9%. The MFC/IC ratio was determined and the fungicidal effect was considered if the MFC value was equal to or 4 times greater than the IC for each isolate.^29^

#### Biofilm Cells

The activity of LQA_78 or AMB was evaluated on sessile and dispersed cells from mature biofilms. Yeasts of *C. gattii* (R265 (NA), R265 (A), and L354) were standardized to 1 × 10^7^ CFU/mL in RPMI 1640 medium with 0.16 M MOPS (=RPMI). Then, 100 μL was added to the wells of 96-well flat-bottom plates and incubated (without agitation) at 35 °C for 48 h for biofilm formation.^30^ After incubation, the dispersed cells present in the supernatant were collected to assess the susceptibility test to LQA_78 or AMB using the broth microdilution assay. The sessile cells were washed twice with PBS and treated with LQA_78 or AMB for 48 h at 35 °C (without agitation). Then, the wells were washed with PBS and the metabolic activity of the biofilm cells was determined by the 2,3-bis (2-methoxy-4-nitro-5-sulfophenyl)-2*H*-tetrazolium-5-carboxanilide (XTT, Sigma-Aldrich) reduction method.^31^ The lowest concentrations inhibiting 50% and 90% (BIC_50_ and BIC_90_, respectively) of fungal growth of dispersed cells and metabolic activity of sessile cells from biofilms were determined.

#### Morphotypes Titan and Enlarged Capsule Cells

The antifungal effect of LQA_78 was also assessed on two morphotypes of *C. gattii* [R265­(NA), R265­(A), and L373], normal cells with enlarged capsule (NcC) and titan cells (Tc), using the broth microdilution assay to compare IC and MFC values with those of normal cells (Nc). The Nc cells were obtained after growth on SDA for 72 h at 37 °C. Nc were then cultivated in an artificial cerebrospinal fluid (ACSF) for 72 h at 37 °C, with agitation at 150 rpm to induce the capsule enlargement (i.e., NcC).^32^ Tc cells (diameter of the cell body ≥10 μm) were obtained by incubating Nc in yeast nitrogen base (YNB, Becton, Dickinson, Sparks, MD, USA) broth for 24 h at 37 °C, with agitation at 150 rpm, adjusting the inoculum to 1 × 10^6^ CFU/mL in PBS plus 10% fetal bovine serum (FBS, Vitrocell, Brazil) and 5% Sabouraud dextrose broth (SDB, Becton, Dickinson, Sparks, MD, USA) incubating in microaerophilia at 37 °C for 72 h with under agitation at 150 rpm.^22,33^

### Action of the Compound LQA_78 on Cell Diameter, Capsule Thickness, and Permeability of *C. gattii*

NcC cells of *C. gattii* R265­(NA), R265­(A), and clinical isolate L373 were washed twice in PBS and the inoculum was adjusted to 1 × 10^7^ CFU/mL in ACSF and treated with LQA_78 (IC_50_ = 2 and IC_90_ = 8 μg/mL) at 35 °C for 48 h. Then, untreated and treated yeasts were washed twice in PBS and a mixture (1:1) of isothiocyanate rhodamine dextran 70 kda (RITC-70kda, Sigma), at 16 μg/mL in ethanol, with Indian ink was studied on a slide to observe the cells by light and fluorescence microscopies for counting and determination of the percentage of cells labeled with fluorochrome.^34^ To measure capsule thickness and body diameter, the ImageJ 1.49v program (https://imagej.nih.gov/ij) was used. Capsule thickness measurement was defined as the difference between the total diameter of the cell (capsule included) and the diameter of the cell body (defined by the cell wall).^23^

### Melanization Assay and Laccase Activity

#### Melanization Assay

The melanization of *C. gattii* strains (R265­(NA), R265­(A), and L373) was induced using minimum medium (15 mM dextrose, 10 mM MgSO_4_, 29.4 mM KH_2_PO_4_, 13 mM glycine, 3 μM thiamine-HCl, with or without 1.5% agar) supplemented with 1 mM L-3,4-dihydroxyphenylalanine (l-DOPA, Sigma-Aldrich).

LQA_78 was added to the medium at concentrations corresponding to the IC_50_ and IC_90_ values to evaluate inhibition of melanin production in inoculated yeasts (20 μL of a suspension containing 1 × 10^6^ CFU/mL). Plates were incubated in a dark, humid chamber at 35 °C for 48–72 h.^35^ *C. albicans* SC5314 was used as a negative control for melanization.

#### Laccase Activity Assay

*C. gattii* yeasts (R265­(NA), R265­(A), and L373) were cultured in 5 mL of yeast extract peptone dextrose at 30 °C for 24 h. Cells were harvested by centrifugation at 1000*g* for 10 min and transferred to 5 mL of asparagine salt medium (0.3% glucose, 0.1% l-asparagine, 0.05% MgSO_4_, 1% Na_2_HPO_4_ [1 M, pH 6.5], 3 μM thiamine, 0.001% CuSO_4_ [0.5 M]). Cultures were incubated for 5 days at 30 °C with shaking at 200 rpm.

After incubation, yeasts were washed with 50 mM Na_2_HPO_4_ (pH 7.0) and adjusted to 1 × 10^7^ CFU/mL in glucose-free asparagine salt medium to induce laccase expression for 72 h at 30 °C under shaking (untreated control). Parallel cultures were treated with LQA_78 at IC_50_ and IC_90_ concentrations under identical conditions.^22^

Laccase activity in the supernatant was normalized to the total number of yeasts at the end of incubation (laccase/yeast). Samples were centrifuged at 4000*g* for 5 min, and 180 μL supernatant was mixed with 20 μL of l-DOPA (10 mM) in 96-well plates (triplicate). Additionally, 450 μL of yeast suspension (treated or untreated; 1 × 10^7^ CFU/mL) was combined with 50 μL of l-DOPA (10 mM) in 24-well plates (duplicate). Supernatant alone (200 μL) and fungal suspension alone (500 μL) were used as blanks, and l-DOPA alone served as the reagent control.

Melanin formation was monitored at 0, 4, 8, 12, and 24 h at 30 °C by measuring optical density at 480 nm. Laccase activity was calculated according to de Sousa et al.^36^

Laccaseactivity(μmol/yeastnumber)=ODmean/(7.9×numberyeastintheculture)

#### Laccase Inhibition Assay

The inhibition assay using commercial laccase isolated from *Trametes versicolor* (Sigma-Aldrich) was performed by incubating 3 μg of laccase dissolved in sterile ultrapure water with the IC_50_ and IC_90_ concentrations of LQA_78 compound and 20 μL of 10 mM l-DOPA. The results were obtained by spectrophotometric reading at 480 nm for a period of 3 h at 30 °C, and the activity of *T. versicolor* laccase under these conditions was determined from the equation described above, replacing the amount of yeast with the mass of protein used.^22^

### Cell Wall Components’ Quantification

For analysis of cell wall components, *C. gattii* R265­(NA) yeasts standardized at 1 × 10^3^ CFU/mL in RPMI medium were exposed to LQA_78 (IC_50_ and IC_90_) for 48 h at 35 °C. Then, the yeasts were washed twice with PBS and fixed with 4% formaldehyde for 30 min, washed in PBS and the yeasts were adjusted to 1 × 10^6^ CFU/mL. Subsequently, the yeasts were labeled with Calcofluor White M2R (25 μg/mL, Sigma-Aldrich), eosin Y (25 μg/mL, Sigma-Aldrich), and aniline blue (25 μg/mL, Sigma-Aldrich), for total quantification of chitin, chitosan, and β-1,3-glucan, respectively.^19^ For each sample 10,000 to 100,000 events were analyzed by flow cytometry using a BD Accuri C6 instrument (Becton, Dickinson, Sparks, MD, USA). The data were processed using BD Accuri C6 software.

### Time-Kill Curve

Nc yeasts from *C. gattii* R265 (NA) standardized at 5 × 10^4^ CFU/mL were exposed to increasing concentrations of LQA_78 or AMB, ranging from 0.5× IC_50_ to 16× IC_50_, in RPMI medium at 35 °C. After different times of incubation, samples were serially diluted (1:10) and plated on SDA, followed by incubation for 48 h at 35 °C. Colony counts were used to calculate log CFU/mL values and to construct the time-kill curves.^37^

### Membrane Permeability Analysis

Nc yeasts of *C. gattii* R265 (NA), standardized at 1 × 10^7^ CFU/mL, were exposed to LQA_78 or AMB at concentrations corresponding to IC_50_, 2× IC_50_, 4× IC_50_, and 8× MC_50_, diluted in PBS and incubated at 35 °C. Untreated yeasts were included as a control for cell membrane integrity. After 1, 8, and 24 h of incubation, the cells were collected by centrifugation at 13,000*g* for 8 min, and the supernatants were analyzed using a spectrophotometer (NanoDrop 2000, Thermo Scientific) to quantify DNA (260 nm) and protein (280 nm) release.^38^

### Cell Death Experiments

*C. gattii* R265­(NA) standardized to 1 × 10^7^ CFU/mL was exposed to LQA_78 (2× IC_50_ to 8× IC_50_), AMB (1 μg/mL), or H_2_O_2_ (50 mM), diluted in PBS, for 24 h at 35 °C. Then, the yeasts were collected, washed with PBS, adjusted to 3 × 10^6^ CFU/mL, and labeled with 5 μg/mL rhodamine 123 (Sigma-Aldrich) (mitochondrial membrane potential) or 40 μg/mL of 2′7′ dichlorofluorescin diacetate (DCFH-DA, Sigma-Aldrich) (reactive oxygen species (ROS) production) for 30 min and at room temperature in a dark chamber.^39^ For DNA fragmentation, cells were fixed, permeabilized with 0.1% Triton X-100 for 30 min at room temperature, and subjected to TUNEL assay (deoxynucleotidyl transferase-mediated BrdUTP labeling), with an APO-BrdU TUNEL assay kit (Invitrogen, Eugene, OR, USA), according to the manufacturer’s protocol. For each sample 10,000 to 100,000 events were analyzed by flow cytometry using a BD Accuri C6 instrument (Becton, Dickinson, Sparks, MD, USA). The data were processed using BD Accuri C6 software, all experiments were run three times, in triplicate.

For chromatin condensation and lipid peroxidation analysis, yeasts were treated and processed as mentioned above and stained with 1 μg/mL Hoechst 33342 (Invitrogen, Eugene, OR, USA) or 1 μM BODIPY 581/591 C11­(Thermo Scientific), respectively, for 10 and 30 min. For chromatin condensation, fluorescence was measure with excitation/emission settings of 350/461 nm and for lipid peroxidation with excitation/emission settings of 485/530 nm (oxidized form, green) and 565/590 nm (reduced form, red) using a BioTek Synergy H1 microplate reader (Agilent). Lipid peroxidation was expressed as the ratio of green to red fluorescence intensities. Experiments were run three times, in triplicate.

To assess Annexin V/propidium iodide (PI) staining, *C. gattii* R265 (NA) yeasts were grown on SDA for 48 h at 35 °C. The inoculum was adjusted to 1 × 10^7^ CFU/mL in PBS in the presence or absence of LQA_78 (16 or 32 μg/mL) or AMB (1 μg/mL) and incubated for 24 h at 35 °C. Cells were centrifuged for 10 min at 8500*g* and washed once with 50 mM Tris–HCl (pH 7.4). Yeasts were then resuspended in 100 mM Tris–HCl containing 10 mM dithiothreitol and incubated for 10 min at 35 °C with shaking at 150 rpm. Subsequently, 1.2 M sorbitol was added at a 3:1 ratio (3 mL sorbitol per 1 mL yeast suspension). Cells were centrifuged again for 10 min at 8500*g* and resuspended in zymolyase buffer (*per* 10 mL: 20 mM potassium phosphate buffer [KH_2_PO_4_/K_2_HPO_4_], 1.2 M sorbitol, and 6 mg zymolyase 20T; pH 7.2; Seikagaku Corp., Tokyo, Japan). Suspensions were incubated for 1 h at 30 °C with shaking at 150 rpm to obtain spheroplasts.

Spheroplasts were washed once with 1.2 M sorbitol^40^ and stained with Annexin V-Alexa Fluor 488 and PI (both Invitrogen, Eugene, OR, USA) according to the manufacturer’s instructions. Briefly, cells were adjusted to 1 × 10^6^ cells/mL in Annexin V binding buffer (10 mM HEPES, 140 mM NaCl, 2.5 mM CaCl_2_, 1.2 mM sorbitol, pH 7.4). Aliquots of 100 μL were transferred to new tubes and 5 μL of Annexin V-Alexa Fluor 488 and PI (5 μg/mL) were added. Samples were incubated for 30 min at room temperature in the dark. Cells were then washed with binding buffer, and 20 μL aliquots were mounted on glass slides and examined using a fluorescence microscope (EVOS FL, Thermo Fisher Scientific). Experiments were performed in biological duplicates and at least 100 cells were analyzed for the following labeling: unlabeled cells, AnnexinV+/PI–, AnnexinV–/PI+, and AnnexinV+/PI+ for determination of percentages.^41^

### Glutathione, Thiol, and Superoxide Dismutase Assay

For quantification of glutathione (GSH), thiol, and activity of superoxide dismutase enzyme of *C. gattii* R265 (NA), yeasts were standardized to 1 × 10^7^ CFU/mL and exposed to LQA_78 (16 or 32 μg/mL) in PBS for 24 h at 35 °C. After this period, cells were lysed according to Cox et al.^42^ and supernatants were collected to perform the analysis.

GSH quantification and superoxide dismutase activity were measured by colorimetric assays (Cayman Chemical, 703002 and Cayman Chemical, 706002, respectively) and thiol quantification was measured by fluorometric assay (Sigma-Aldrich, MAK151), according to the manufacturer’s instructions. All assays were read in the same microplate reader mentioned above in biological and technical duplicates. Values were determined according to the standard curve provided by each assay.

### Antifungal Activity in an *In Vivo* Model of *Galleria mellonella*

#### Antifungal Effect

For determining the antifungal activity, *G. mellonella* larvae (1.5 to 2 cm, average weight of 150 mg) were infected with 10 μL of *C. gattii* R265­(NA) yeasts (5 × 10^8^ CFU/mL) in the left hemocoel, corresponding to a final inoculum of 5 × 10^6^ CFU *per* larvae. After 30 min, 10 μL of LQA_78 (3 μg/larva or 6 μg/larva, corresponding to 20 mg/kg or 40 mg/kg, respectively), AMB (0.75 μg/larva, corresponding to 5 mg/kg), or combinations of LQA_78 and AMB (3 μg/larva and 0.75 μg/larva, respectively) were injected into the right hemocoel, and PBS was injected into the infected larvae (untreated group). All injections were performed using a 10 μL Hamilton syringe to ensure accurate inoculum. The larvae (*n* = 20 *per* group) were incubated at 37 °C for 7 days. The survivors and their health status were assessed daily to construct the survival and morbidity curves, respectively.^43,44^ Hemolymph from untreated larvae and larvae treated with LQA_78 alone or combined with AMB for 24 h postinfection (*n* = 4 *per* group) was also collected and added to a tube containing an equal volume of IPS (Insect Physiological Saline) buffer.^43^ The hemolymph was serially diluted, plated on SDA, and incubated for 48 h at 35 °C for CFU counting. This assay was performed twice.

#### Total Hemocytes in the *G. mellonella* Hemolymph

The larvae were separated into seven groups (*n* = 4 larvae/group): (i) noninjected group, i.e., noninfected and nontreated larvae; (ii) PBS, which received 10 μL of PBS twice; (iii) untreated group, which received 10 μL of *C. gattii* R265 (5 × 10^8^ CFU/mL) and 10 μL of PBS; (iv) LQA_78 only, that received 10 μL of 20 mg/kg LQA_78 and 10 μL of PBS; (v) R265+LQA_78 20 mg/kg, that received 10 μL of *C. gattii* R265 (5 × 10^8^ CFU/mL) and 10 μL of 20 mg/kg LQA_78; vi) R265+LQA_78 40 mg/kg, that received 10 μL of *C. gattii* R265 (5 × 10^8^ CFU/mL) and 10 μL of 40 mg/kg LQA_78; and vii) R265+LQA_78+AMB, that received 10 μL of *C. gattii* R265 (5 × 10^8^ CFU/mL) and 10 μL of 20 mg/kg LQA_78 combined with 5 mg/kg AMB. Thirty minutes elapsed between the injections in the right and left hemocoels. After 24 h, the larvae hemolymph was collected as described above, and total hemocytes were counted in a Neubauer chamber. This assay was performed twice.

## Results

### LQA_78 Shows Antifungal Activity on *C. gattii* Strains

Seven *C. gattii* strains were subjected to antifungal susceptibility testing to determine the minimum inhibitory concentrations (MICs) and the MFCs of AMB, 5-FC, FLC, and LQA_78. AMB and 5-FC presented MIC of 0.03–0.25 μg/mL and 0.5–1 μg/mL, respectively, while FLC and the compound LQA_78 showed inhibitory activities in a concentration range from 2 to 32 μg/mL (Table 1). The fungicidal effect was observed for AMB (MFC: 0.25–1 μg/mL) and LQA_78 (MFC: 16–64 μg/mL), while FLC (MFC ≥ 16 μg/mL) and 5-FC (MFC > 64 μg/mL) were considered fungistatic for the most isolates tested (see MFC/IC ratio in Table S1). Among the clinical isolates, only L186 showed a resistance profile to FLC, according to the previously demonstrated epidemiological breakpoints.^28^ In addition, we obtain modal MIC values of 1 μg/mL for FLC and 0.5 μg/mL for AMB against *C. parapsilosis* ATCC 22019 quality control strain; the expected values are described in the CLSI documents, ensuring the reliability of results of antifungal susceptibility testing.

**Table 1:** Susceptibility of *Cryptococcus gattii* Strains to Amphotericin B (AMB), Fluconazole (FLC), 5-Flucytosine (5-FC), and the Organoselenium LQA_78^a^ ^,^ ^b^

strains	AMB			FLC			5-FC			LQA_78		
	IC_50_	IC _90_	MFC	IC _50_	IC _90_	MFC	IC _50_	IC _90_	MFC	IC _50_	IC _90_	MFC
R265(NA)	0.03	0.06	0.25	4	16	32	1	2	>64	2	8	16
R265(A)	0.03	0.06	0.25	16 ^R^	32	>64	1	2	>64	2	8	16
L373	0.25	0.5	1	4	8	16	1	4	>64	16	32	64
L186	0.125	0.25	0.25	16 ^R^	32	>64	1	2	>64	8	16	32
L508	0.06	0.125	0.25	4	8	32	0.5	4	>64	16	32	64
525	0.25	0.25	0.25	4	4	16	0.5	2	>64	4	4	16
527	0.25	0.25	0.5	2	4	16	0.5	2	>64	2	4	16

a Values are expressed in μg/mL.

b IC_50_: the lowest concentration that inhibits 50% of fungal growth. IC_90_: the lowest concentration that inhibits 90% of fungal growth. MFC: lowest concentration that kills >99.9% of yeasts. R: resistant. R265­(NA): TBZ Nonadapted Strain.^23^ R265­(A): TBZ Adapted Strain.^23^

At this point, we choose three representative strains (R265 NA, R265 A, and clinical isolate L373) to perform next assays for investigating the inhibition of virulence factors and mechanisms of fungal death induced by the compound.

### LQA_78 Has an Antifungal Effect on Morphotypes and Biofilm Dispersed Cells of *C. gattii*

Both morphotypes cells with enlarged capsule (NcC) and titan cells (Tc) of *C. gattii* R265­(NA), *C. gattii* R265 (A), and clinical isolate L373 were inhibited by AMB and FLC, with ICs and MFC values similar to those found for normal cells (Nc) (Table 2). AMB was able to inhibit the growth by 90% with concentrations between ≤0.03 and 1 μg/mL and MFC ranging from 0.06 μg/mL to 2 μg/mL. FLC inhibited growth between 4 and 16 μg/mL in R265­(NA) and in the TBZ-adapted strain R265­(A), an increase in IC values from 16 to 64 μg/mL was observed, higher than the IC values found for the NA strain, as expected. LQA_78 was also able to inhibit the growth of normal cells and Tc and NcC morphotypes of *C. gatti* R265 (NA and A) and L373 with concentrations ranging between 2 and 32 μg/mL and maintained the MFC values from 16 to 64 μg/mL (Table 2).

**Table 2:** Susceptibility of Morphotypes from *Cryptococcus gattii* R265 (NA and A) and Clinical Isolate L373 to Amphotericin B (AMB), Fluconazole (FLC), and Compound LQA_78^a^ ^,^ ^b^

		AMB			FLC			LQA_78		
strains/morphotypes		IC_50_	IC_90_	MFC	IC_50_	IC_90_	MFC	IC_50_	IC_90_	MFC
R265(NA)	Nc	0.03	0.06	0.25	4	16	32	2	8	16
	NcC	≤0.03	≤0.03	0.125	4	16	64	1	4	16
	Tc	≤0.03	≤0.03	0.06	4	16	64	2	4	16
R265(A)	Nc	0.03	0.06	0.25	16	64	>64	2	8	16
	NcC	0.06	0.125	0.5	32	64	>64	8	32	64
	Tc	0.06	1	2	16	64	>64	8	16	32
	Nc	0.25	0.5	1	4	8	16	16	32	64
L373	NcC	0.125	1	2	2	4	16	1	8	16
	Tc	0.25	0.5	1	2	4	16	4	8	16

a Values are Expressed in μg/mL.

b IC_50_: lowest concentration that inhibits 50% of fungal growth. IC_90_: lowest concentration that inhibits 90% of fungal growth. MFC: lowest concentration that kills >99.9% of yeasts. Nc: normal cells, NcC: enlarged capsule cells, and Tc: titan cells.

LQA_78 was able to inhibit growth of dispersed cells of *C. gattii’s* biofilm at 8 μg/mL in both R265 strains (NA and A), but it was not able to inhibit growth of the dispersed cells from the clinical isolate L373. On sessile cells, LQA_78 did not show inhibition on the concentrations tested. AMB was used as a control and was able to inhibit growth of biofilm dispersed cells and reduce metabolic activity of sessile cells (Table 3).

**Table 3:** Antifungal Activity of Amphotericin B (AMB) and the Compound LQA_78 on Sessile Cells and Dispersed Cells from Mature Biofilms of *Cryptococcus gattii* R265­(NA), R265­(A), and L373^a^ ^,^ ^b^

strains	sessile cells				dispersed cells			
	AMB		LQA_78		AMB		LQA_78	
	BIC_50_	BIC_90_	BIC _50_	BIC_90_	BIC_50_	BIC_90_	BIC_50_	BIC_90_
R265(NA)	0.03	0.03	>128	>128	0.03	0.06	8	>128
R265(A)	0.03	0.03	>128	>128	0.03	0.06	8	>128
L373	0.25	1	>128	>128	0.06	1	>128	>128

a Values are expressed in μg/mL.

b BIC_50_: lowest concentration that inhibits 50% of the metabolic activity of sessile or dispersed cells from biofilms. BIC_90_: lowest concentration that inhibits 90% of metabolic activity of sessile or dispersed cells from biofilms.

### The Capsule Permeability of *C. gattii* Is Affected by LQA_78

Due to the importance of the capsule as a virulence factor in *C. gattii* yeasts,^40–42^ it was evaluated whether the compound LQA_78 has some action on the capsule of *C. gattii* strains. Treatment with LQA_78 (IC_50_ = 2 μg/mL and IC_90_ = 8 μg/mL) did not change the capsular thickness (Figure 2A-C). However, the compound was able to increase the capsular permeability of approximately 50% of NcC from all *C. gattii* strains after 48 h of treatment, while untreated NcC had less penetration of the RITC-70kda, demonstrating lower permeability in its capsule (Figure 2D–F).

**Figure 2:**
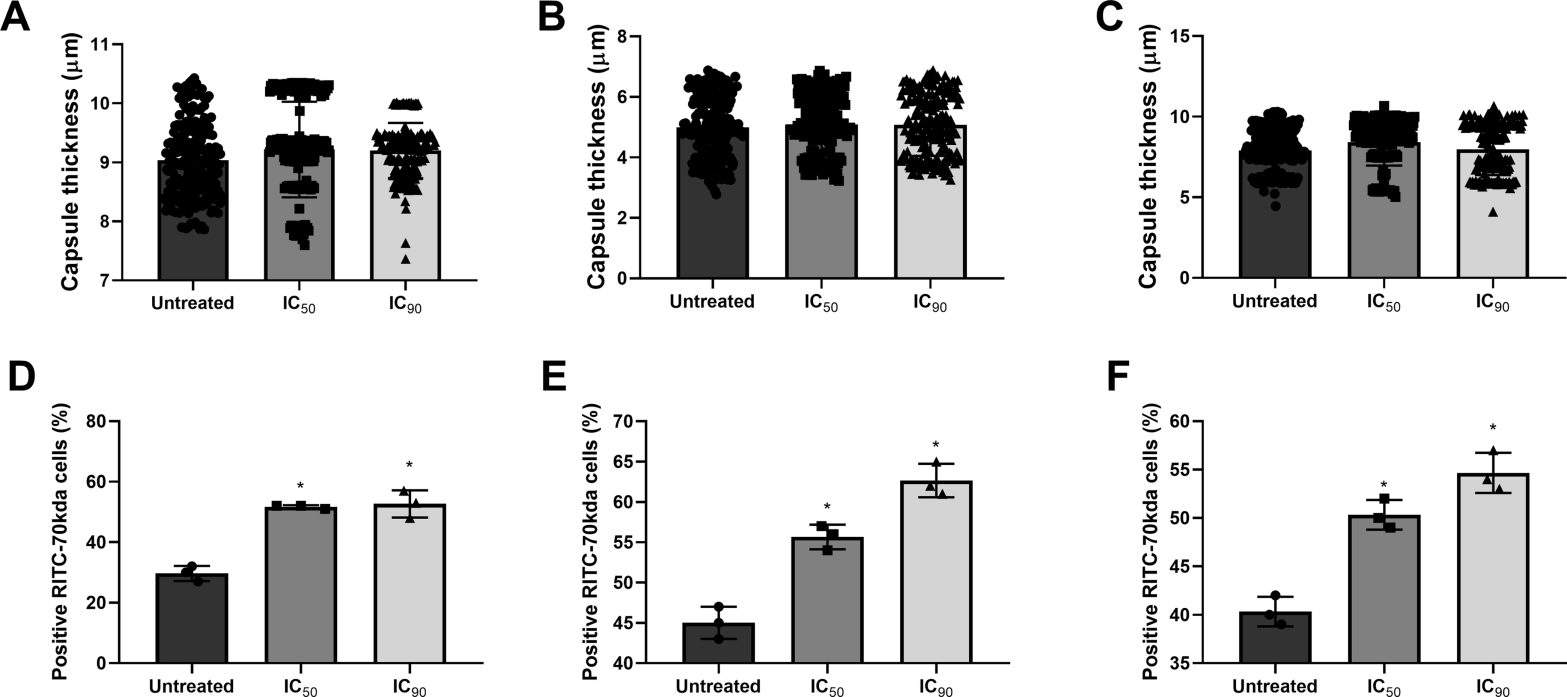
Capsular morphometry and permeability of yeasts with the enlarged capsule (NcC) of *Cryptococcus gattii* after exposure to the compound LQA_78. Action of compound LQA_78 at 2 and 8 μg/mL (IC_50_ and IC_90_, respectively) on capsule size (A–C) and capsule permeability (D–F) of NcC at 1 × 10^7^ UFC/mL of *C. gattii* R265NA (left graphs), R265A (middle graphs), and L373 (right graphics) in LCR medium for 48 h at 35 °C. After this period, the yeasts were stained with India ink to count cells and measure capsular thickness and stained with RITC-70kda fluorophore for analysis of capsule permeability. *p < 0.05 when compared to the untreated group (one-way ANOVA with Dunnett’s test, three independent experiments, *n* = 100 cells).

### LQA_78 Inhibits Melanization and Decreases Laccase Activity of *C. gattii*

LQA_78 inhibited melanization at concentrations ≥ IC_90_ values in all tested strains. At 2 μg/mL, a reduction in pigmentation was already evident in *C. gattii* R265­(NA) colonies (Figure 3). A similar inhibitory pattern was detected in the TBZ-adapted strain (R265A) and in the clinical isolate L373; however, the degree of melanization varied among strains. Notably, strains with higher melanin production required higher concentrations of LQA_78 to achieve inhibition, whereas L373 displayed lower baseline melanization even in the absence of the compound.

**Figure 3:**
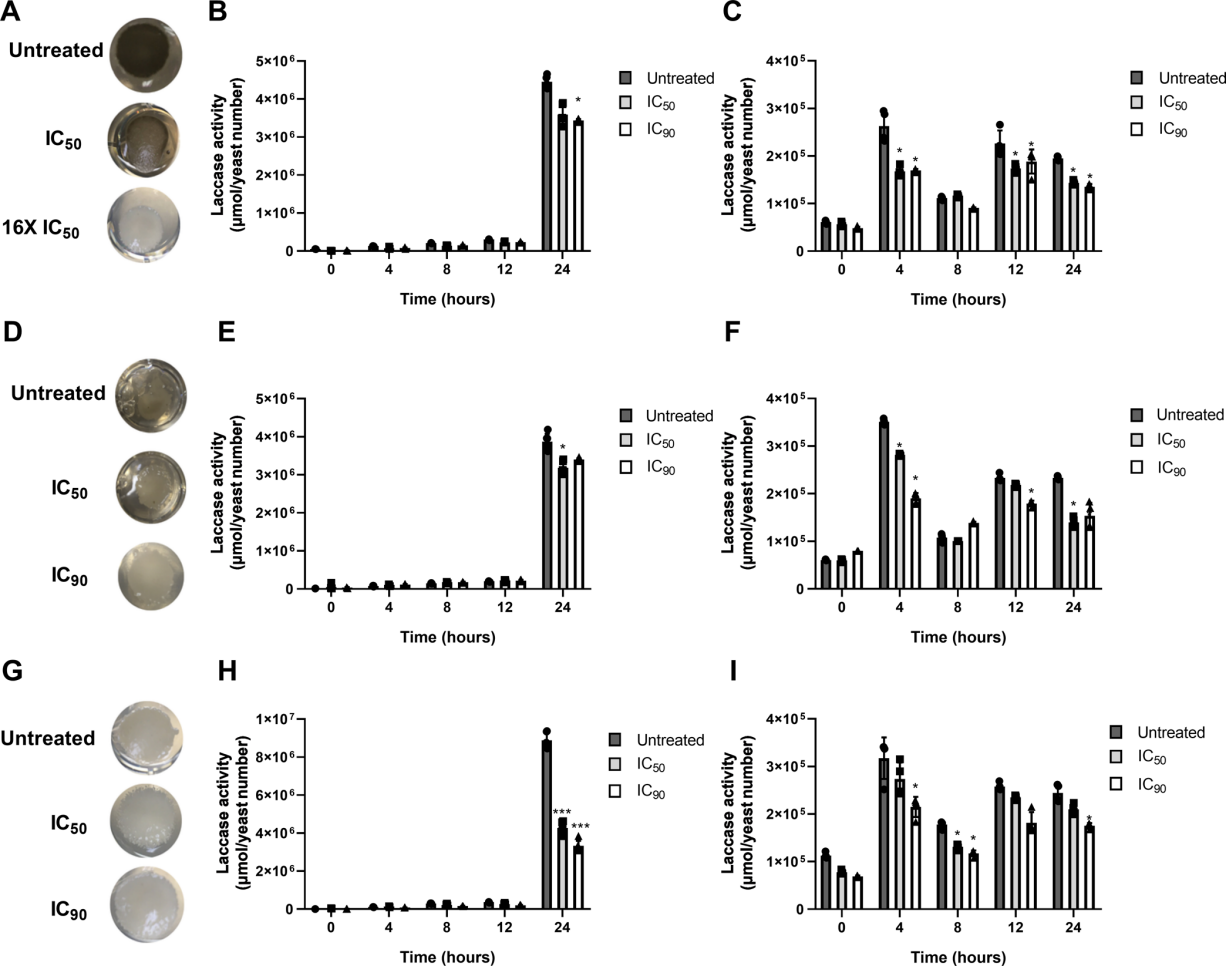
Effect of compound LQA_78 on melanization and on laccase activity of *Cryptococcus gattii*. For melanization analysis (A,D,G), *C. gattii* strains (R265­(NA), R265­(A), and L373) were standardized to 1 × 10^6^ CFU/mL and cultivated on minimal medium agar containing l-DOPA and LQA_78 for 72 h at 35 °C. *Candida albicans* SC5314 was included as a negative control for melanization (data not shown). Laccase activity was assessed in culture supernatants (B,E,H) and yeast cells (C,F,I) at 0, 4, 8, 12, and 24 h of incubation at 30 °C, by measuring optical density at 480 nm. A-C: R265 (NA) strain (IC_50_ = 2 μg/mL), D-F: R265­(A) strain (IC_50_ = 2 μg/mL), and G-I: clinical isolate L373 (IC_50_ = 4 μg/mL). The IC_90_ value for all strains was 8 μg/mL. *p < 0.05 and ***p < 0.001 compared to untreated controls (Two-way ANOVA with Tukey’s multiple comparison test, three independent experiments performed in triplicate for supernatants and duplicate for yeasts).

The decrease of the laccase activity by the LQA_78 was also observed after yeast treatment in the laccase-inducing medium. It was possible to observe that both IC_50_ and IC_90_ values were capable of inhibiting laccase activity (Figure 3). In the yeast supernatant, the activity of the enzyme was mostly visualized after 24 h of incubation with l-DOPA and this behavior was observed in all strains tested (Figure 3B, E, H). In the yeasts, the laccase activity may be detected from initial hours to 24 h of incubation with l-DOPA; then, the inhibitory activity of the compound LQA_78 was significant on the laccase enzyme into yeasts (Figure 3C, F, I). Given that melanin is deposited in the fungal cell wall, we also quantified β-1,3-glucan, chitin, and chitosan but observed no significant changes following LQA_78 treatment (Figure S1). In addition, the commercial laccase isolated from *T. versicolor* was treated with the IC_50_ and IC_90_ values of LQA_78, but no reduction in the laccase activity was observed when compared to the untreated samples, suggesting that the compound did not directly inhibit the enzyme (Figure S2).

### LQA_78, at Higher Concentrations, Inhibits and Reduces Cell Viability by Increasing Plasma Membrane Permeability

After treatment with the compound LQA_78, it was observed that in the initial 12 h there was a slight reduction in cell viability of *C. gattii* R265 (NA) exposed to 8x and 16x IC_50_ (IC_50_ = 2 μg/mL) remaining until 48 h of incubation, while 4x IC_50_ inhibited up to 24 h and after this period the fungus returned to growth (Figure 4A). On the other hand, the standard antifungal AMB led cells to fast loss of cell viability with 1 h at highest fungicidal concentrations (Figure 4B) as shown in previous studies.^45^

**Figure 4:**
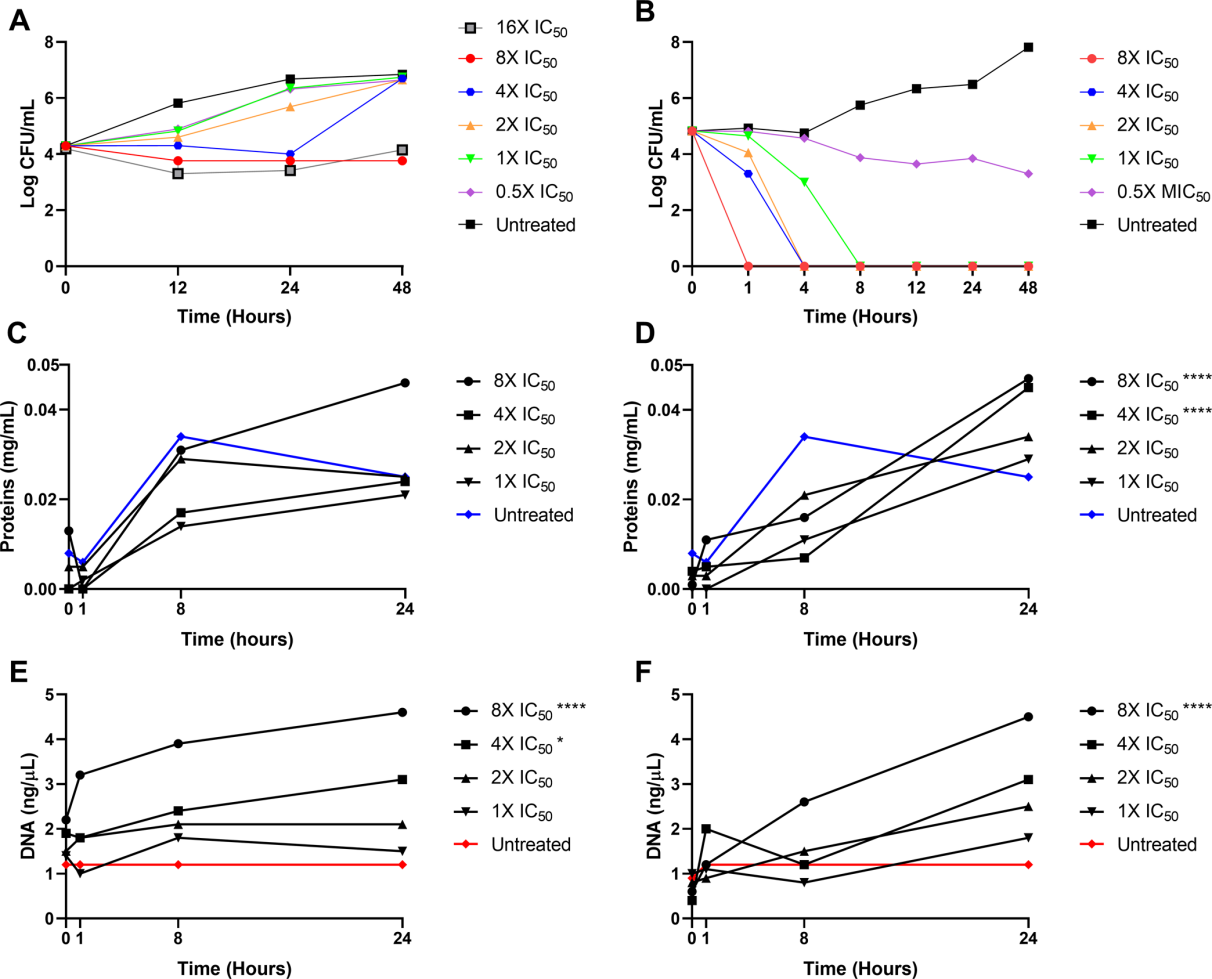
Time-kill curves and plasma membrane permeability of *Cryptococcus gattii* R265 (NA) yeasts treated with LQA_78 or AMB. Yeasts at 5 × 10^4^ CFU/mL were exposed to LQA_78 (left column; IC_50_ = 2 μg/mL) or AMB (right column; IC_50_ = 0.03 μg/mL) at concentrations ranging from 0.5x to 16x IC_50_ in RPMI 1640 buffered with 0.16 M MOPS at 35 °C for 48 h. Panels A, C, and E correspond to LQA_78, while panels B, D, and F correspond to AMB. Time-kill curves (A,B) were generated by serially diluting samples at different times of incubation, plating on SDA, and determining Log CFU/mL. Membrane permeability was assessed by quantifying proteins (C,D) and DNA (E,F) released into the supernatant at 0, 1, 8, and 24 h, using absorbance at 280 and 260 nm, respectively. *p < 0.05 and ****p < 0.0001 when compared with untreated control (Two-way ANOVA, Dunnett’s test, two independent experiments, performed in duplicate).

Cytoplasmic membrane permeability was observed after LQA_78 treatment at concentrations above 4xIC_50_ in *C. gattii* R265 (NA) yeasts during the 24 h incubation period, leading to the release of DNA and/or proteins into the medium (Figure 4C,E). The same behavior was seen in yeasts treated with AMB, an antifungal that increases the plasma membrane permeability resulting in a fungicidal effect (Figure 4D,F).

Based on the results observed here, LQA_78 concentrations of 16 and 32 μg/mL were selected for subsequent experiments to further characterize the cell mechanisms involved in growth inhibition and fungal death.

### *C. gattii*’s Redox System Is Disturbed after LQA_78 Treatment Inducing Oxidative Stress

LQA_78 enhanced ROS in *C. gattii* when treated with 32 μg/mL (Figure 5A) and induced a higher level of lipid peroxidation at both tested concentrations (16 and 32 μg/mL) (Figure 5B), suggesting that higher concentrations of the organoselenium lead *C. gattii* to an oxidative stress contributing to cell membrane damage.

**Figure 5:**
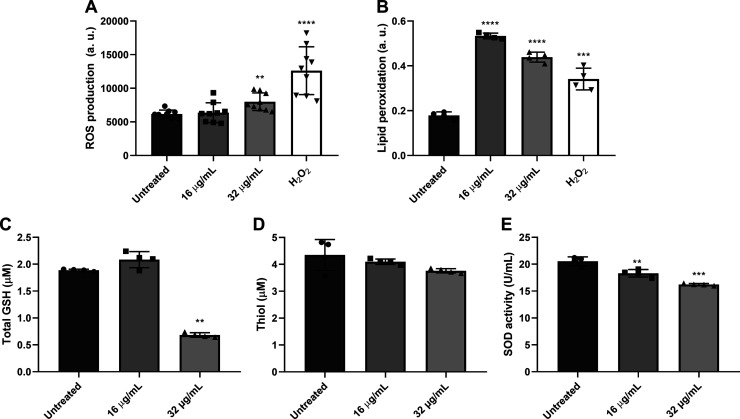
LQA_78 induces ROS accumulation disrupting redox system homeostasis of *Cryptococcus gattii*. Productions of ROS (A), lipid peroxidation (B), intracellular GSH levels (GSH, C), total thiols (D), and superoxide dismutase activity (SOD, E) were evaluated in *C. gattii* R265 (NA) yeasts treated with higher concentrations of LQA_78 (16 and 32 μg/mL) for 24 h at 35 °C. ROS production was assessed by flow cytometry using DCFH-DA staining, and lipid peroxidation was quantified by fluorometric analysis using BODIPY C11. GSH, thiols, and superoxide dismutase activity were determined according to the manufacturer’s instructions. a.u., arbitrary unit. **p < 0.01, ***p < 0.001, and ****p < 0.0001 compared with untreated control (one-way ANOVA, Dunnett’s test, three independent experiments performed in triplicate (A) and two independent experiments, performed in duplicate for (B–E)).

Since LQA_78 induced oxidative stress in *C. gattii*, we next asked whether disruption of GSH-dependent redox homeostasis underlies this phenotype. Black and colleagues (2024) recently demonstrated that the glutathione-mediated redox system has significant influence in many *Cryptococcus* virulence factors. Our results revealed that at 32 μg/mL, LQA_78 led to depletion of intracellular GSH concentration (Figure 5C) and reduced activity of the superoxide-dismutase enzyme (Figure 5E), which could explain the ROS accumulation detected in *C. gattii* yeasts treated with LQA_78. Total thiols were not significantly decreased overall, although a modest reduction was apparent at the highest concentration of the compound (Figure 5D).

### LQA_78 Induces *C. gattii* Death by Apoptosis and Necrosis

To determine the type of cell death triggered by LQA_78 on *C. gattii*, we followed the guidelines proposed by Carmona-Gutierrez et al.^41^ assessing mitochondrial membrane potential (Figure 6A), chromatin condensation (Figure 6B), DNA fragmentation (Figure 6C), together with annexin V and PI labeling for phosphatidylserine (PS) exposition and plasma membrane permeability, respectively (Figure 6D,E). LQA_78, at higher concentrations (16 and 32 μg/mL), reduced the mitochondrial membrane potential, increased chromatin condensation and DNA fragmentation, which are common markers of apoptotic processes. Similar data were observed in annexin V+/PI– and annexin V+/PI+ labeling in yeasts exposed to 16 μg/mL of LQA_78, indicating an induction of cell death by apoptosis and necrosis, respectively. In comparison, AMB treatment resulted in a high proportion of both annexin V and PI positive cells, and a minor proportion of annexin V positive cells, indicating the loss of plasma membrane integrity and a predominance of cell death by necrosis.

**Figure 6:**
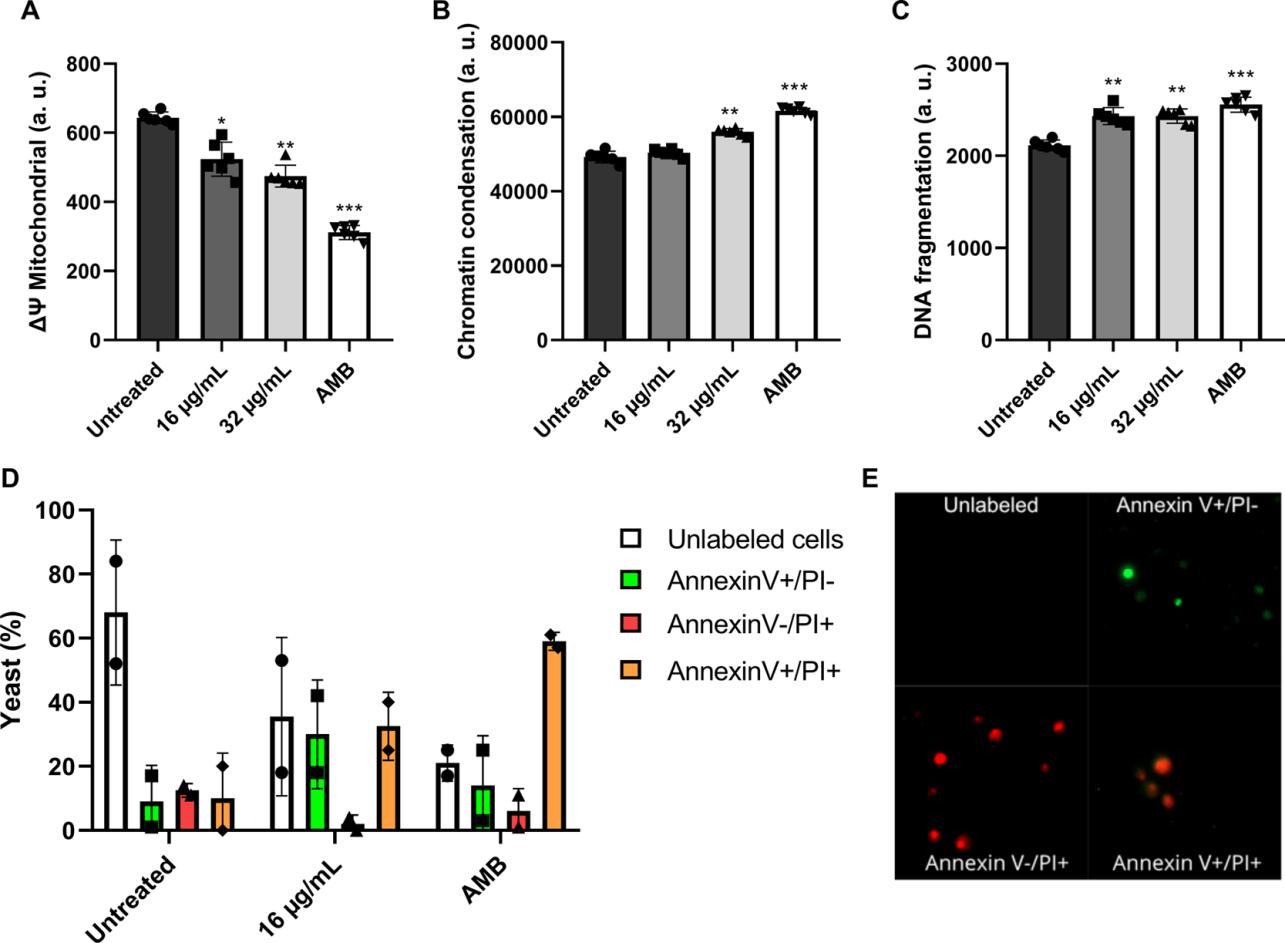
Analysis of cell death of *Cryptococcus gattii* induced by LQA_78 at higher concentrations. *C. gattii* R265 (NA) yeasts treated with LQA_78 (16 and 32 μg/mL) or amphotericin B (AMB at 1 μg/mL) for 24 h at 35 °C were analyzed by flow cytometry or microscopy after fluorescent labeling for mitochondrial membrane potential (Rhodamine 123) (A), chromatin condensation (Hoechst 33342) (B), DNA fragmentation (TUNEL) (C), and exposition of phosphatidylserine (PS) at the cytoplasmic membrane (Annexin V) and membrane permeability (Propridium iodide, PI) (D,E). Hydrogen peroxide (H_2_O_2_) and AMB were used as controls for the assays. a.u., arbitrary unit. *p < 0.05, **p < 0.005, and ***p < 0.001 when compared to untreated yeast (one-way ANOVA with Dunnett’s test, two independent experiments, performed in triplicate) (A–C), and Annexin V/PI assay was performed in two independent experiments where 100 cells were analyzed in each experiment (D,E).

### LQA_78 Has Antifungal Efficacy in *G. mellonella* Larvae Infected by *C. gattii*, Reducing the Fungal Burden and Promoting an Increase in Hemocyte Count.

As demonstrated by De Jesus et al.,^22^ LQA_78 does not present toxicity at doses below 46.5 mg/kg in *G. mellonella* larvae; therefore we employed the *in vivo* invertebrate model for fungal infection using *C. gattii* R265 (NA) yeasts. Treatment with LQA_78 alone at doses of 20 or 40 mg/kg, as well as AMB (5 mg/kg), significantly reduced the larval mortality (Figure 7A) and morbidity (Figure 7B). Combined treatment of LQA_78 (20 mg/kg) with AMB (5 mg/kg) also resulted in a significant reduction in morbidity and lethality of larvae infected by *C. gattii*. In addition, fungal burden in the larval tissue was significantly reduced after monotherapy or combined therapy (Figure 7C). However, no additional benefit over either monotherapy was observed (Figure 7A–C).

**Figure 7:**
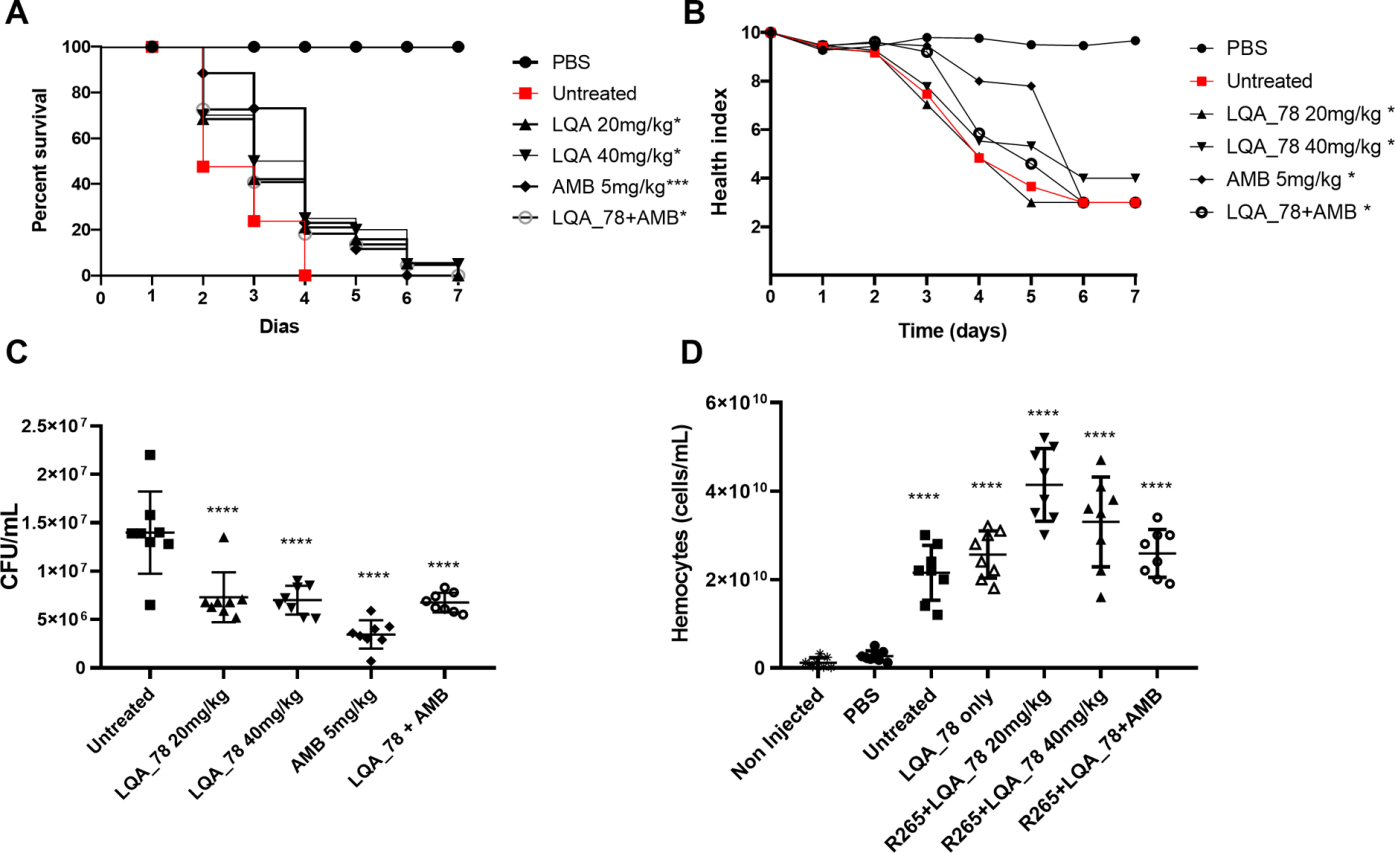
Antifungal activity of the compound LQA_78 in the *Galleria mellonella* larvae infected with *Cryptococcus gattii* yeasts. (A,B) Efficacy of LQA_78 treatment in monotherapy and in combined therapy with AMB on larvae infected with *Cryptococcus gattii* R265 yeasts (NA) by analyzing the health index (A) and survival curve (B) (*n* = 40 larvae/group). *p < 0.05 and ***p = 0.0001 when compared with the untreated group, Kaplan–Meier curve and Mantel–Cox Log–Rank test for survival data and two-way ANOVA with multiple comparisons test for health index. (C) CFU/mL count in hemolymph 24 h after infection (*n* = 8 larvae/group). ****p < 0.001 when compared with the untreated group by the one-way ANOVA, with Dunnett’s post test. (D) Total hemocyte count 24 h after infection and/or treatment infection (*n* = 8 larvae/group). ****p < 0.001 when compared with the noninjected group by one-way ANOVA, with Dunnett’s post test.

It was also verified that healthy larvae treated with LQA_78 as well as larvae infected and treated with LQA_78 had a higher number of total hemocytes when compared to larval groups uninfected and noninjected larvae, and uninfected larvae that received PBS injection (PBS) (*p* < 0.0001, Figure 7D). In addition, we observed that the LQA_78 treatment of infected larvae increased the hemocyte number compared with other larval groups (Figure 7D), which may be beneficial for larvae to effectively combat *C. gattii* infection together with the LQA_78 antifungal action.

## Discussion

*C. gattii* is a *Cryptococcus* species responsible for causing cryptococcosis in health individuals. Although *C. neoformans* is the most prevalent species in terms of epidemiology and higher mortality rate, *C. gattii* shows its relevance since it is well-known that species have different ways of establishing infection in the host and responding to the presence of antifungals. It is suggested that *C. gattii* yeasts present additional processes to evade the host’s immune system,^46^ such as the reduction of Th1 and Th17 responses in the lung tissue of immunocompetent individuals^11^ and a greater expression of the laccase enzyme, capable of attenuating the Th17 type response and increasing the survival of *C. gattii* yeasts in macrophages when compared to *C. neoformans*.^47^ Additionally, during clinical diagnosis, the *Cryptococcus* species are often not differentiated, highlighting the need to provide antifungals that act on both *C. neoformans* and *C. gattii.* Our group has previously described LQA_78, a synthetic organoselenium compound with inhibitory action against *C. neoformans* isolates at concentrations of 2 to 64 μg/mL, in addition to presenting a fungicidal effect and the ability to inhibit virulence factors such as capsule size and melanin production.^22^ Here, we tested LQA_78 against *C. gattii* isolates, evaluated its mechanisms inducing fungal cell death and antifungal effectiveness using cryptococcosis model in invertebrates to further demonstrate LQA_78 potential as an antifungal compound.

Corroborating the data mentioned above, the compound was able to inhibit the growth of *C. gattii* yeasts at concentrations ranging from 2 to 32 μg/mL, values similar to those obtained for *C. neoformans*. Additionally, there was no change in the IC_50_ and IC_90_ values of LQA_78 when compared both R265 strains, adapted and nonadapted to TBZ. It is known that the MIC of clinical azole agents (such as FLC) on strains of *C. neoformans* and *C. gattii* adapted to TBZ is higher in relation to nonadapted strains. Bastos et al. demonstrated that the excessive use of antifungal pesticides such as TBZ lead to cross-resistance between those used in agronomy and those used in the clinical treatment of cryptococcosis, becoming a critical point when we talk about antifungal resistance;^23^ so it is important to search for antifungals that do not get affected by this factor. The fungicidal effect was also observed after subculturing the cells from LQA_78 treatments in the broth microdilution test, with an MFC value of 16 μg/mL in both strains of *C. gattii* R265 (NA and A). Although the compound was not efficient in reducing the metabolic activity of sessile mature biofilm cells, the importance of eradicating dispersed cells stands out due to its ability to increase the spread of infection and production of new biofilms.^48^ Therefore, despite having obtained higher ICs, the compound was able to inhibit dispersed cells and prevent their growth, which could be useful to control infection.

In addition to the results obtained with tolerant strains, interesting data were also seen in the LQA_78 susceptibility of *C. gattii* morphotypes of R265­(NA), R265­(A), and clinical isolate L373. The presence of titan cells is a powerful cryptococcal tool in the progress of fungal infection, since the hosts’ macrophages are unable to phagocytose them due to their large cell diameter. The presence of dense and thicker capsules also helps these yeasts to resist oxidative stress produced by macrophages.^49^ LQA_78 was able to inhibit the growth of titan cells and increased capsule cells of *C. gattii* strains at concentrations between 1 and 8 μg/mL.

The *C. gattii* capsule is one of its greatest virulence factors, interfering with the host’s immune response and the action of conventional antifungals, so, we evaluated whether the compound LQA_78 would be able to reduce the capsule thickness, as seen in the *C. neoformans*.^22^ However, the compound did not show this activity in *C. gattii* at inhibitory concentrations. Despite this, when treated with LQA_78, NcC from *C. gattii* showed an increase in the capsule permeability. So, even without reducing the thickness of the capsule, the compound was able to increase its permeability, weakening a major virulence factor of this yeast.

*C. gattii* is more frequently associated with pulmonary infection, with studies reporting lung involvement in approximately 60% of cases, and this pulmonary incidence has also been observed in murine models.^1,50,51^ In this context, *C. gattii* has been associated with increased capsule thickness and a higher propensity for titan cell formation in lungs, particularly among hypervirulent strains. Capsule enlargement and titan cell formation is regulated by environmental sensing and genetic pathways, including quorum-like signaling and transcriptional regulation that control morphological transitions and capsule remodeling.^52^ However, these features are strain-dependent and influenced by environmental and host conditions, making it difficult to define representative average values.^53–55^ Importantly, despite these advances, the precise mechanisms underlying the increased propensity of *C. gattii* to form titan cells remain not fully understood.

Beyond the capsule and titan cells, *C. gatiii* shares other major virulence factors with *C. neoformans* species, as shown in our previous work. As demonstrated by De Jesus et al.^22^ in *C. neoformans*, LQA_78 was able to inhibit melanin production partially or totally in both strains of *C. gattii* R265 (NA and A) and the clinical isolate. The production of melanin by *Cryptococcus* is a major protective mechanism for yeasts, protecting them from UV radiation, oxidative stress, immune system cells, and antifungal action.^11,56,57^ It is not common for standard antifungals to present this mechanism of action; therefore, this finding suggests that LQA_78 could make the fungal cell more vulnerable by inhibiting melanin production by the l-DOPA pathway. This finding can be justified by the ability of the compound LQA_78 to also decrease the laccase activity, responsible for the conversion of l-DOPA into melanin, during induction, which also suggests that the compound may cause some interference in the expression of genes linked to this enzyme, such as the *LAC1* and *MPK1*.^58^ It is known that laccase activity is significantly higher in *C. neoformans* and its melanization occurs faster when compared to *C. gattii*,^59^ affecting its virulence during infection.^60^ Our data corroborate those of de Souza et al.,^59^ as it was observed that *C. gattii* R265 needed 72 h to melanize in solid medium, while *C. neoformans* H99 melanization may be observed at 48 h.^22^ The laccase inhibition by the compound also stands out because the enzyme showed to be necessary to inhibit recruitment of neutrophils and the expression of pulmonary chemokines in the initial phase of the *C. gattii* infection.^47^ This fact has a high impact, as it is known that the *C. gattii* species is known to be capable of subverting the action of the host’s immune system to cause infection in immunocompetent individuals.^46^ Even though cryptococcal cell wall components are also important to modulate immune response from host and prevent antifungal entrance^61^ by retaining melanin,^62^ our findings demonstrated that LQA_78 did not appear to affect the production of β-1,3-glucan, chitin, and chitosan.

The time-kill curve showed that LQA_78 slightly reduced the viability of *C. gattii* R265 cells in the initial 12 h of treatment, even at higher concentrations of 16 and 32 μg/mL. Given the fact that in this assay the inoculum used (5 × 10^4^ CFU/mL) has a higher yeast concentration than the inoculum used in the susceptibility tests (1 × 10^3^ CFU/mL), we suppose that the inoculum size is fundamental for the fungicidal activity of the compound LQA_78. Corroborating the time-kill curve, the plasma membrane permeability analysis data demonstrated loss of integrity of the fungal cell membrane in the initial 12 h of treatment with the compound, promoting the extravasation of DNA and proteins, especially at higher tested concentrations. Interestingly, a distinct response was observed when compared with our previous findings in the *C. neoformans* H99 strain.^22^ Although *C. neoformans* H99 and *C. gattii* R265 exhibited similar MIC values for LQA_78, the two species displayed markedly different responses to the compound. In *C. neoformans*, treatment with LQA_78 at 16 and 32 μg/mL resulted in complete loss of viability, with no detectable CFU after 24 and 4 h of treatment, respectively, indicating the fungicidal potential of the compound. In contrast, although LQA_78 promoted an initial reduction in the viability of *C. gattii* R265 cells and induced plasma membrane damage, fungal regrowth was observed after prolonged incubation. These findings suggest that *C. gattii* R265 exhibits a reduced susceptibility to the fungicidal effects of LQA_78 when compared with *C. neoformans* H99, highlighting species-specific differences in the response to this compound.

When it comes to mechanism of cell death, we adopted the guidelines proposed by Carmona-Gutierrez et al.^41^ to identify if *C. gattii* yeasts were undergoing necrosis or apoptosis when interacting with higher concentrations of LQA_78 that promoted viable cell reduction and increased membrane permeability. Our results revealed that cells treated with 16–32 μg/mL provided evidence of cell events associated with death, since we could visualize increased DNA fragmentation and chromatin condensation, decreased mitochondrial membrane potential, and ROS accumulation. Therefore, to confirm this hypothesis annexin V/PI staining was assessed, commonly used to distinguish apoptotic and necrotic cells. Finally, our results demonstrated that cells treated with 16 μg/mL of LQA_78 were mostly positive for apoptosis and necrosis markers. Although there are no studies attesting that AMB leads *Cryptococcus* to death from apoptosis, it is known that at lower concentrations an apoptosis-like phenotype can be observed, while higher concentrations of AMB induce a necrosis-like phenotype since it leads to an oxidative burst and increased PI uptake.^63^ In this regard, our data corroborate those of the previous work, indicating that 1 μg/mL AMB triggers a response mediated by oxidative stress, which progresses to necrosis after prolonged exposure.

As well as the ebselen molecule, LQA_78 also has a selenium atom. Thangamani and colleagues demonstrated that ebselen exhibited antifungal activity by reducing the amount of intracellular GSH in fungal cells, leading to an increase in ROS production.^64^ The possible mechanism of action of ebselen was suggested based on the fact that when *Candida* spp. and *Cryptococcus* spp. were cultivated in medium containing inhibitory concentrations of ebselen and supplemented with GSH, their growth was reestablished, even in the presence of the compound that has antifungal activity on both species. It has been shown that ebselen reacts with exposed cysteines to form covalent selenosulfide adducts and inhibit the thioredoxin reductase of *Escherichia coli* and *A. fumigatus* ^65,66^ and it involves the formation of a covalent bond between the Se atom and the active site of cysteines.^66^ So, considering that LQA_78 shares with ebselen the Se atom and also leads to ROS production, we hypothesize that both compounds may share this mechanism of action. Our results corroborated with Thangamani’s data, showing that LQA_78 at higher concentrations also depletes intracellular GSH concentrations, leading to increase of ROS produced by *C. gattii* yeasts and decreases the activity of the superoxide dismutase enzyme, also responsible for regulating oxidative stress.^64^ As discussed by Black et al., maintenance of redox homeostasis is essential for *Cryptococcus* virulence.^67^ It has been demonstrated that thiols such as GSH have a direct and indirect modulatory role in melanin production, iron acquisition, titan cell production and defense against oxidative stress delivery by the host. Mutant strains lacking *GSH* genes were less virulent than wild-type strains of *C. neoformans*, indicating that the redox system plays a fundamental role in terms of virulence and pathogenicity of *Cryptococcus*.^68^ Collectively, these results highlighted the potential of redox homeostasis as a therapeutic target in antifungal strategies and further substantiate the evidence regarding the activity of the organoselenium LQA_78.

Finally, we evaluated the efficacy of the compound LQA_78 in an *in vivo* *G. mellonella* model of *C. gattii* infection. LQA_78, at doses of 20 mg/kg and 40 mg/kg, combined or not with AMB (5 mg/kg), was able to reduce the fungal infection increasing the larval lifespan when compared to untreated larvae. Additionally, the antifungal therapies reduced fungal load in the larval tissue. Furthermore, we also visualized that treatment with LQA_78 in both infected and uninfected larvae demonstrated a certain increase on the number of hemocytes in the larval hemolymph. Furthermore, as mentioned, since the host’s immune response plays a key role to fight *C. gattii* infection, our findings lead to the need to further investigate the immunomodulation and antifungal activity of LQA_78 in a murine model of infection.

Data from this study have shown that LQA_78 has an inhibitory effect on *C. gattii* growth and virulence factors such as capsule, melanin production, and laccase activity. The LQA_78 compound at a higher concentration was able to induce death mediated by apoptosis and necrosis in *C. gattii* yeasts by starting an oxidative stress, associated with a decrease of GSH levels and superoxide dismutase activity. Finally, LQA_78 demonstrated an important antifungal effect *in vivo* using a larva model of *G. mellonella*. These findings provide evidence that organoselenium compounds, such as LQA_78, could be a promising strategy to approach antifungal therapy, presenting notable inhibitory and fungicidal activity against *Cryptococcus* species.

## Supplementary Material



## Data Availability

All data will be made available from the corresponding author upon request.

## Notes

MFC/IC ratios obtained for amphotericin B (AMB), fluconazole (FLC), 5-flucytosine (5FC), and LQA_78 against *C. gattii* reference strains and clinical isolates; effect of LQA_78 on the cell wall components of *C. gattii* R265 and effect of LQA_78 on the enzymatic activity of laccase from *T. versicolor* (PDF)
