# Supplementary material for: An Organoselenium Compound Induces Death in Cryptococcus gattii Yeast by Apoptosis and Necrosis and Shows Antifungal Efficacy in Galleria mellonella
Source: ACS Omega. 2026 Jul 14;11(29):43540–54. doi: 10.1021/acsomega.6c02274 (PMC13425516; doi:10.1021/acsomega.6c02274)
Supplement: Supplementary file 1 [file ao6c02274_si_001.pdf]

Supplementary information

Title: An organoselenium compound induces death in *Cryptococcus gattii* yeast by apoptosis and necrosis and shows antifungal efficacy in *Galleria mellonella*

Running title: Selenium compound activity against *Cryptococcus gattii*

Authors: Letícia Serafim da Costa[1], Daniel Felipe Freitas de Jesus[1], Larissa Dunkl Brujas[1], Isadora Maria de Oliveira[2], Rafael Wesley Bastos[3,6], Analy Salles de Azevedo Melo[4], Daniel Assis Santos,[5,6] Hélio A. Stefani[2], and Kelly Ishida[1#]

[1]Department of Microbiology, Institute of Biomedical Sciences, University of São Paulo (USP), Sao Paulo, Brazil
[2]Department of Pharmacy, School of Pharmaceutical Sciences, University of São Paulo (USP), São Paulo, Brazil
[3]Center of Biosciences, Federal University of Rio Grande do Norte (UFRN), Natal, Brazil
[4]Department of Medicine, Federal University of São Paulo (UNIFESP), São Paulo, Brazil
[5]Institute of Biomedical Sciences, Federal University of Minas Gerais (UFMG), Belo Horizonte, Brazil
[6]Brazilian National Institute of Science and Technology in Human Pathogenic Fungi (INCT-FUNVIR), São Paulo, Brazil

#Address correspondence to Kelly Ishida: ishidakelly@usp.br

Address: Prof. Lineu Prestes Avenue, 1374, ICB II Building - Laboratory 150, Department of Microbiology of the Institute of Biomedical Sciences, University of São Paulo, 05508-000, São Paulo, São Paulo, Brazil

Table S1. MFC/IC ratios obtained for amphotericin B (AMB), fluconazole (FLC), 5-flucytosine (5FC), and LQA_78 against *Cryptococcus gattii* reference strains and clinical isolates.

Strains	AMB	FLC	5-FC	LQA_78
	MFC/IC_{90}	MFC/IC_{50}	MFC/IC_{50}	MFC/IC_{90}
R265(NA)	4	8	>64	2
R265(A)	4	>4	>64	2
L373	2	4	>64	2
L186	1	>4	>64	2
L508	2	4	>128	2
525	1	2	>128	4
527	2	8	>128	4

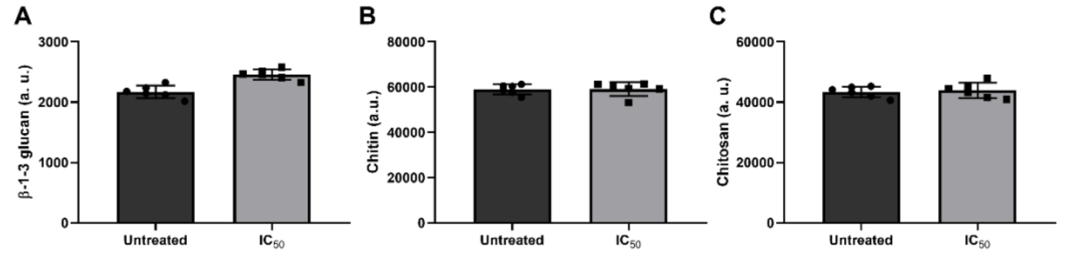

Figure S1. Effect of LQA_78 on the cell wall components of *Cryptococcus gattii* R265. (A) quantification of β-1,3-glucan, (B) chitin, and (C) chitosan. Yeasts treated with the IC_{50} of LQA_78 for 48 h were analyzed by flow cytometry using aniline blue (25 µg/mL, Sigma-Aldrich) to quantify β-1,3-glucan, Calcofluor White M2R (25 µg/mL, Sigma-Aldrich) to quantify chitin, and eosin Y (25 µg/mL, Sigma-Aldrich) to quantify chitosan. a.u. = arbitrary units.

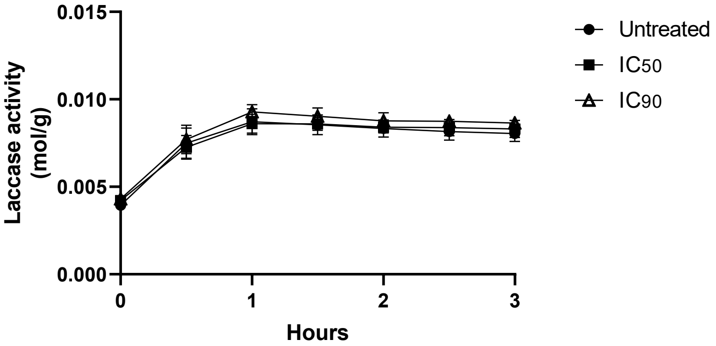

Figure S2. Effect of LQA_78 on the enzymatic activity of laccase from *Trametes versicolor.* Commercial laccase from *T. versicolor* was incubated with LQA_78 at IC_{50} and IC_{90} concentrations and 10 mM L-DOPA. Enzymatic activity was determined by measuring melanin formation resulting from L-DOPA oxidation at 480 nm over 3 h at 30 °C. Data are presented relative to the untreated control.
